# Sera contributing to mycobacterial growth restriction *in vitro* display enhanced Fc-mediated phagocytosis

**DOI:** 10.1016/j.isci.2025.112504

**Published:** 2025-04-23

**Authors:** Krista E. van Meijgaarden, Patricia S. Grace, Wenjun Wang, Delia Goletti, Fabrizio Palmieri, Manfred Wuhrer, Tom H.M. Ottenhoff, Simone A. Joosten

**Affiliations:** 1 Leiden University Center for Infectious Diseases (LUCID), Leiden University Medical Center, Leiden, the Netherlands; 2 Ragon Institute of MGH, MIT, and Harvard, Boston, MA, USA; 3 Harvard T.H. Chan School of Public Health, Boston, MA, USA; 4 Center for Proteomics and Metabolomics, Leiden University Medical Center, Leiden, the Netherlands; 5 Department of Epidemiology and Preclinical Research, National Institute for Infectious Diseases, Rome, Italy; 6 Clinical Department, National Institute for Infectious Diseases, Rome, Italy

**Keywords:** Microbiology, cell biology

## Abstract

Tuberculosis (TB) remains a major cause of global mortality. Understanding the underlying immune response to the pathogen, *Mycobacterium tuberculosis (Mtb)*, is essential for the development of vaccines. Evidence is accumulating supporting a contribution of innate immune cells and antibodies in *Mtb* control. Here, we focus on the functional capacity of antibodies from individuals with TB disease, both before and after TB treatment, individuals with TB infection, and healthy uninfected individuals, using an adapted *in vitro* mycobacterial growth inhibition assay, measuring Bacillus Calmette-Guérin **(**BCG) growth inhibition. Sera displayed heterogeneous impact on mycobacterial growth control. This was correlated with enhanced phagocytic capacity, which was abrogated by blocking Fc receptors (FcR) and depletion of antibodies. This phenotype negatively associated with mono- and digalactosylated Fc-glycans of IgG. Together, we demonstrate disease state independent direct effects of sera to mycobacterial growth control and antibody-FcR interactions modulating phagocytic capacity.

## Highlights

• Antigen-specific antibody responses are not different across TB disease states
• Sera can affect mycobacterial growth control *in vitro*
• FcR expression profiles are heterogeneous and impact BCG growth control
• Phagocytosis as Fc-mediated effector function contributes to mycobacterial growth control

## Graphical abstract

**Figure:**
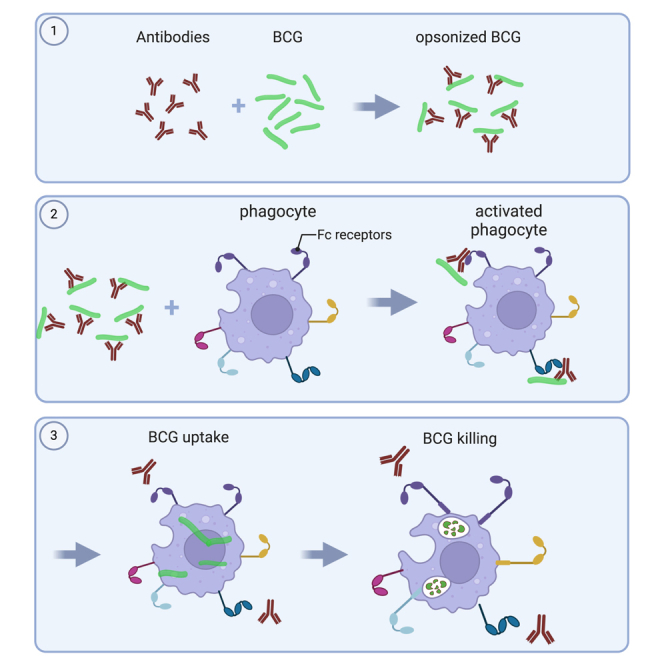

## Introduction

Tuberculosis (TB) remains a global threat and the major cause of death by a single infectious disease, only surpassed by COVID-19 during the pandemic. TB incidence has increased to over 10 million cases, and TB deaths to 1.3 million in 2022 alone.^1^ Traditionally, protection against TB is considered to be T cell mediated, as the pathogen *Mycobacterium tuberculosis* (*Mtb*) resides intracellularly, but over the last decade the role of B cells and antibodies in early stages of infection as well as during active TB disease has been investigated.^2^^,^^3^ One of the first, although disputed, indications was derived from *Mtb*-infected B cell knock-out mice, which displayed increased bacterial burdens in the lung, increased inflammatory cytokines and tissue neutrophilia.^4^^,^^5^^,^^6^ This could be reversed by adoptive transfer of naive B cells.^4^^,^^5^ In addition, adoptive transfer of serum from infected mice reversed the TB-associated neutrophilia and inflammation as reflected by IL-17-producing cells, indicating a role for antibodies in acute infection.^4^^,^^5^^,^^6^^,^^7^ In non-human primates, a model more closely resembling human disease, analysis of individual lung granulomas during the acute phase of infection showed increased bacterial burden upon B cell depletion.^8^^,^^9^ This, however, varied greatly within and between infected animals, and within the individual granulomas, suggesting a modulating pro-inflammatory role for B cells in the local response.^9^ Furthermore, in individuals with TB disease, B cells were functionally impaired in proliferation, antibody, and cytokine production compared to treated, latently infected or healthy controls (HCs), while these functions were restored following adequate treatment.^10^

In TB, antigen-specific antibodies have been detected in individuals with TB disease or latent infection, and recently Fc-features, such as isotype and glycosylation, of these antibodies have been linked to distinct functions that may contribute to infection control.^11^^,^^12^ Relationships between disease states and different antibody subclasses are exemplified by a study in HIV-infected individuals which showed a tendency for decreased purified protein derivative of *Mtb* (PPD)-specific IgG3 levels associated with recurrent TB^13^ and by an Italian cohort where IgG4 levels in individuals with active TB disease were increased compared to individuals infected with TB (TBI) and those successfully treated for TB disease.^14^ Additionally, the inflammatory environment in TB strongly influences post translational modifications and in particular variations in the glycan structure affect the functional capacity of antibodies, adding to the complexity of their functional assessment in TB.^15^ Glycosylation profiles of IgG and Fc domains, but not Fab fragments, discriminate between TBI and TB disease with e.g., increased IgG galactosylation in TBI and increased IgG fucosylation in individuals with TBI at risk to progress to TB disease.^14^^,^^16^^,^^17^ Finally, PPD-specific digalactosylated IgG antibodies were the best biomarker to discriminate between TB disease and TB infection despite significant heterogeneity within groups possibly reflecting the spectrum of disease.^11^^,^^16^

Antibodies are important in modulating the immune response during other infectious disease^18^; they facilitate immune effector functions, including phagocytosis, resulting in subsequent antigen processing and presentation, complement-mediated lysis, and NK cell activation through interactions of antibody Fc with Fc receptors (FcR) expressed on the surface of NK cells as well as monocytes and macrophages.^19^^,^^20^^,^^21^ Studies on antigen-specific (monoclonal) antibodies assessed specificity against cell wall associated oligosaccharides like (lipo)arabino mannan ([L]AM), PPD and some (secreted) mycobacterial proteins like antigen 85 (Ag85), ESAT-6, heparin binding hemagglutinin (HBHA), α-crystallin or the phosphate transporter subunit protein PstS1. HBHA-specific antibodies isolated from plasma blasts of individuals with TB disease or *Mtb*-exposed healthcare workers controlled bacterial growth in an *in vitro* lung epithelial cell model and were all IgA, not IgG antibodies.^22^ Watson et al. isolated human monoclonal antibodies, directed against two PstS1 epitopes, with antimicrobial activity in an FcR-dependent manner in mice.^23^ Similarly, antibodies against AM enhanced FcγR-mediated *Mtb* phagocytosis and reduced intracellular growth in THP-1 macrophages, mediated by phago-lysosomal fusion.^24^ Furthermore, bacterial burden was reduced in FcγR-humanized mice upon transfer of these AM antibodies.^24^^,^^25^^,^^26^ In an additional study of TB patient sera, while TB-specific phagocytic function was similar across patients with TBI or TB disease, TBI patients possessed PPD-specific IgG levels with higher affinity for FcγRIIIa which was associated with enhanced antimicrobial functions, such as NK cell-mediated antibody-dependent cellular cytotoxicity and degranulation.^11^ Together these data indicate that several functions mediated by antibodies may influence TB disease control in patients.

In this study we focus on the functional contribution of antibodies to mycobacterial control, and the possible mechanisms thereof, using an adapted version of the mycobacterial growth inhibition assay (MGIA). We show that *in vitro* growth control can be modulated by antibodies that display enhanced Fc-mediated phagocytic effector function.

## Results

### Antigen-specific antibody responses are not different across TB disease states

**Figure 1:**
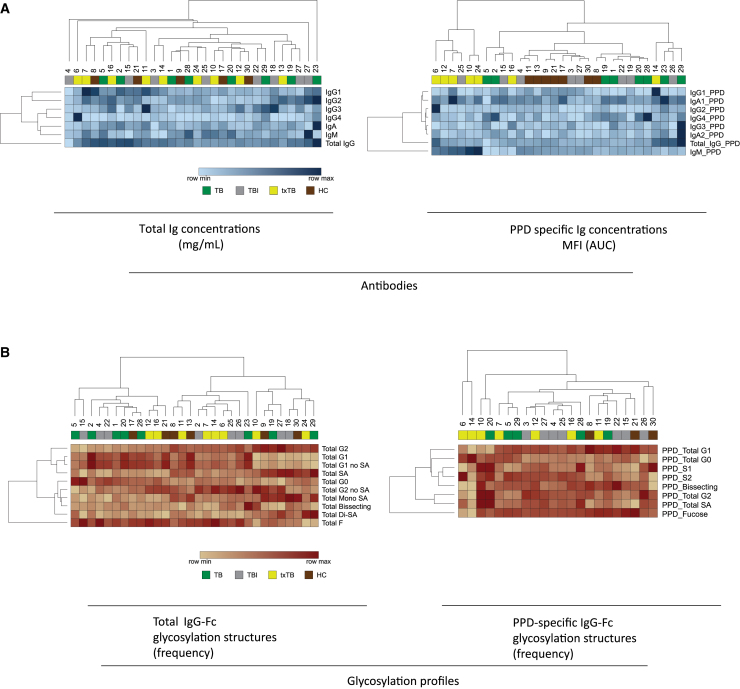
Antibody quantification and characterization of sera Heatmaps were created using hierarchical clustering on rows and columns with Euclidean distance and average linkage method with resulting dendrograms using Morpheus software (Broad Institute). Color scaling is relative within each row and each heatmap includes a color-coded row indicating the individual sera (numbers) and TB disease state with TB patients in green, TBI in gray, treated TB individuals in yellow and healthy controls in brown. (A) Heatmaps indicate total and PPD-specific concentration of antibody subtypes as determined by ELISA (mg/mL) and Luminex (AUC of fluorescence intensity) respectively and (B) glycosylation profiles of total and PPD-specific IgG Fc-regions. PPD-specific glycosylation is missing for sera #1, 2, 9, 13, 17, 18, 23, and 24.

Concentrations of antibodies in serum samples are visualized in heatmaps for total and PPD-specific antibodies (Figure 1A) with (PPD-specific) antibody glycosylation profiles additionally shown in Figure 1B.^14^ Sera were numbered and color-coded based on individual disease state (see legends Figures 1A and 1B). On a subset of patients with sufficient serum, PPD-specific glycosylation analysis was performed. Hierarchical clustering did not result in distinct (antigen-specific) antibody class or subclass profiles in relation to clinical classification, nor did analysis of glycosylation profiles for total IgG-Fc or PPD-specific IgG-Fc(Figure 1B).

### Sera can affect mycobacterial growth control, which correlates with secreted analyte concentrations

**Figure 2:**
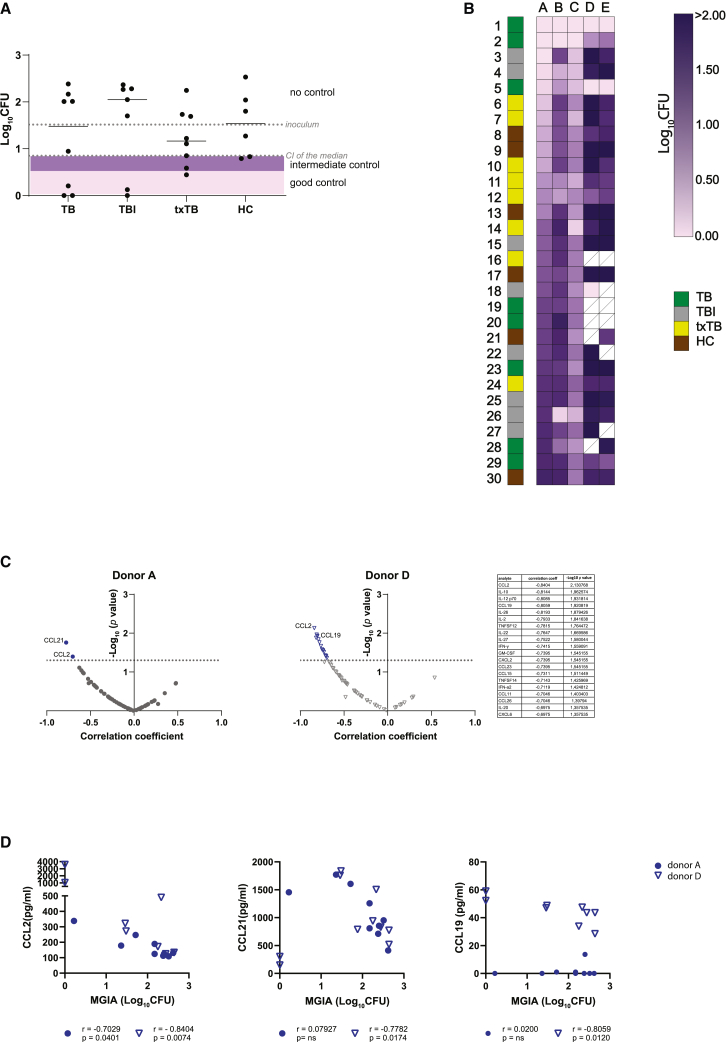
Functional contribution of sera to mycobacterial growth control which correlates with cytokine/chemokine production Live BCG was pre-incubated with different sera before addition to PBMCs, cocultured for 96 h before incubation in MGIT tubes in a BACTEC machine. (A) MGIA result for all sera tested with PBMC A is plotted as scatterplot with median over individual disease grouping, gray dotted lines represent inoculum and lower limit of the confidence interval of the median. Samples with good control of BCG growth are in indicated in the light pink box representing > 1 log CFU reduction, intermediate control is shown in the purple box (growth control between the lower limit of the confidence interval and the 1 log). No significant differences between the groups were identified as tested by Kruskal-Wallis with Dunn’s multiple test correction (B) Summarizing heatmap, color scaling MGIA results as log_10_CFU for all sera included for 5 PBMCs tested in columns, with individual sera in rows. White fields with diagonal line indicate missing datapoints. For sera of 5 TB disease (1, 2, 5, 23, and 29), 4 TB infected (3, 4, 15, and 25), 1 TB treated (10) and 2 healthy individuals (9; 17), supernatants were collected after coculture for 96 h with live BCG and PBMCs (A and/or D) and soluble analytes were measured by Luminex multiplex bead array assays. (C) Spearman’s rank correlation between soluble analytes and MGIA results was determined and plotted per PBMC with a -log_10_(*p* value) on the y axis and the correlation coefficient r on the x axis. In blue, significant correlating analytes are represented. For PBMC D significant analytes are listed in a table. Donor A is shown as dots and donor D as open triangles. (D) Correlations of three example analytes are plotted, CCL2, CCL19 and CCL21 for PBMC A (dots) and PBMC D (open triangles). Correlation coefficient r, and *p* values as tested by Spearman’s rank correlation are shown below each graph.

Sera were assessed for the ability to reduce bacterial burden in an adapted MGIA. Addition of sera contributed to different levels of mycobacterial growth control. All 30 sera included in the study were tested on the same peripheral blood mononuclear cells (PBMC) A, a healthy blood bank donor unrelated to the serum donors, in the same experiment to rule out donor sample and inter assay variability as much as possible. BCG growth control was observed with sera from all clinical groups; good mycobacterial growth control, as defined by a 1 log reduction of colony forming units (CFU) compared to the inoculum, was reached in three individuals with TB disease (1, 2, and 5), two individuals with TBI (3 and 4) and one individual treated for TB (6). Furthermore, sera from two additional individuals treated for TB (7 and 10) and two HCs (8 and 9) showed intermediate growth control, defined by a logCFU below the lower limit of the confidence interval (Figure 2A). Expanding these results further to 5 PBMC donors (A to E) tested for the same 30 sera revealed a pattern in which several sera (1, 2, and 5) performed equally well in all PBMCs used, whereas other sera contributed to more variable growth control (e.g., sera 14 and 26), or no control at all (e.g., sera 29 and 30) (Figure 2B, supplementary datafile). Our screen revealed that antibodies from TB patients could promote mycobacterial growth restriction independent of disease state, including healthy individuals.

Previously, cytokine and chemokine signals have been found to correlate with bacterial control.^27^ Here, MGIA was repeated with a selection of PBMCs (A and D) and sera (TB, *n* = 5; TBI, *n* = 4; txTB, *n* = 1 and HC, *n* = 2) based on observed growth control results and availability. Using multiplex cytokine and chemokine assays, 70 out of 73 unique analytes were detected in supernatants collected after 96 h of coculture. Correlation with the mycobacterial growth control results was assessed (Figure 2C, supplementary data file). Multiple soluble analytes significantly correlated with reduced CFU in MGIA: two analytes, CCL2 and CCL21 for PBMC A and 15 analytes for PBMC D including CCL2 and CCL19. Increased concentrations of cytokines and chemokines correlated with increased BCG growth control (Figure 2D), suggesting a contribution for sera in activation of immune cells.

### FcR expression profiles are heterogeneous and impact BCG growth control

**Figure 3:**
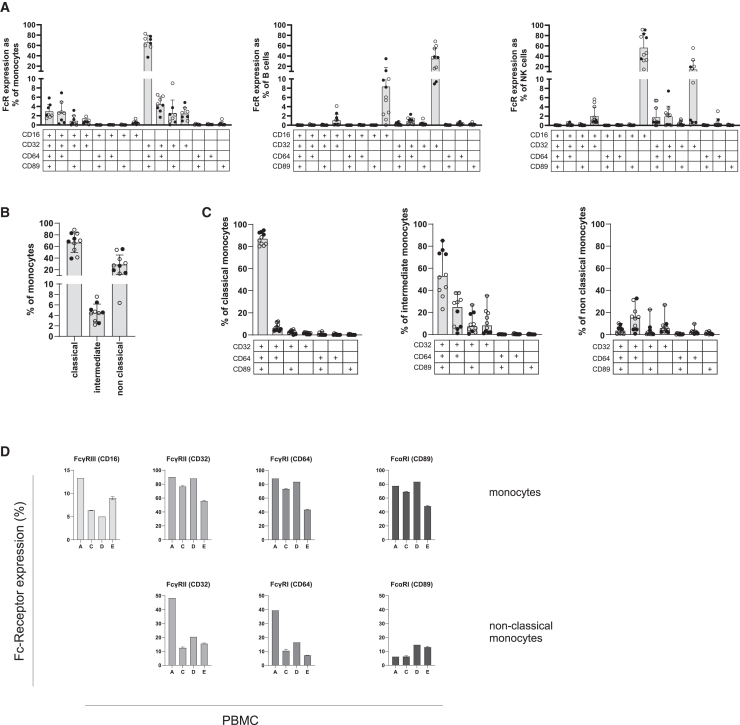
FcR profiles and effect of FcR interaction on mycobacterial growth control (A) FcR expression (CD16, CD32, CD64, and CD89) on monocytes, B cells and NK/NKT cells (n = 8–11 healthy individuals) was assessed by flow cytometry. Boolean gating was applied according to the matrix displayed at the x axis and percentage of positive cells is depicted as bars (median with range) with individual measurements as dots on the y axis. (B) Monocytes are further subtyped into classical, intermediate and non-classical based on their CD14 and CD16 expression. (C) For each monocyte subset Boolean gating for FcRs was applied as in (A). Black dots (A–C) represent the PBMCs used for the MGIA as displayed in Figure 2B. (D) Bar chart (mean with SD) representing the total FcRs expressed on the monocytes of the PBMCs used for MGIA (upper panel) or on non-classical monocytes (lower panel).

Sera are the only variable in the MGIA where immune cell activation was observed. We therefore sought to further characterize interactions between antibodies, as major serum component, and Fc-receptors as their natural ligands on immune cells. FcγRI, II, III, and FcαRI expression on monocytes, B cells and NK cells was measured in PBMCs of 11 anonymous blood bank donors, including PBMCs A, C, D, and E (Figures 3A and S1A). The majority of monocytes, 65%, expressed a combination of FcγRI (CD64), FcγRII (CD32), and FcαRI (CD89) whereas co-expression with FcγRIII (CD16) was only observed in 3% of monocytes. FcγRII on B cells and FcγRIII on NK cells were mostly expressed as a single receptor, contrasting with the combination of FcRs expressed by monocytes. Monocyte subsets were classified based on the CD14 and CD16 expression as classical (CD14^+^CD16^−^; 66%), intermediate (CD14^+^CD16^+^; 4.7%), and non-classical (CD14^−^CD16^+^; 29%) monocytes (Figures 3B and S1B). Co-expression of FcRs was observed on classical monocytes and to a lesser extent on intermediate monocytes where surface expression was more variable (Figure 3C). In total 28% of non-classical monocytes, expressed FcγRII of which 19% also expressed FcγRI. When focusing on the PBMCs used in the MGIA, interindividual FcR differences represent the range of FcR expression observed and were most diverse in non-classical monocytes, a subset previously identified as relevant for mycobacterial growth control.^27^ Comparing PBMC A to E, the lower levels of FcγRI and FcγRII expression in total monocytes PBMC E and the higher surface expression of FcγRI and FcγRII in the non-classical monocytes PBMC A, could reflect the functional difference between PBMC donors as PBMC E was least susceptible to mycobacterial growth modulation by sera (Figure 3D).

### Phagocytosis as Fc-mediated effector function contributing to mycobacterial growth control

**Figure 4:**
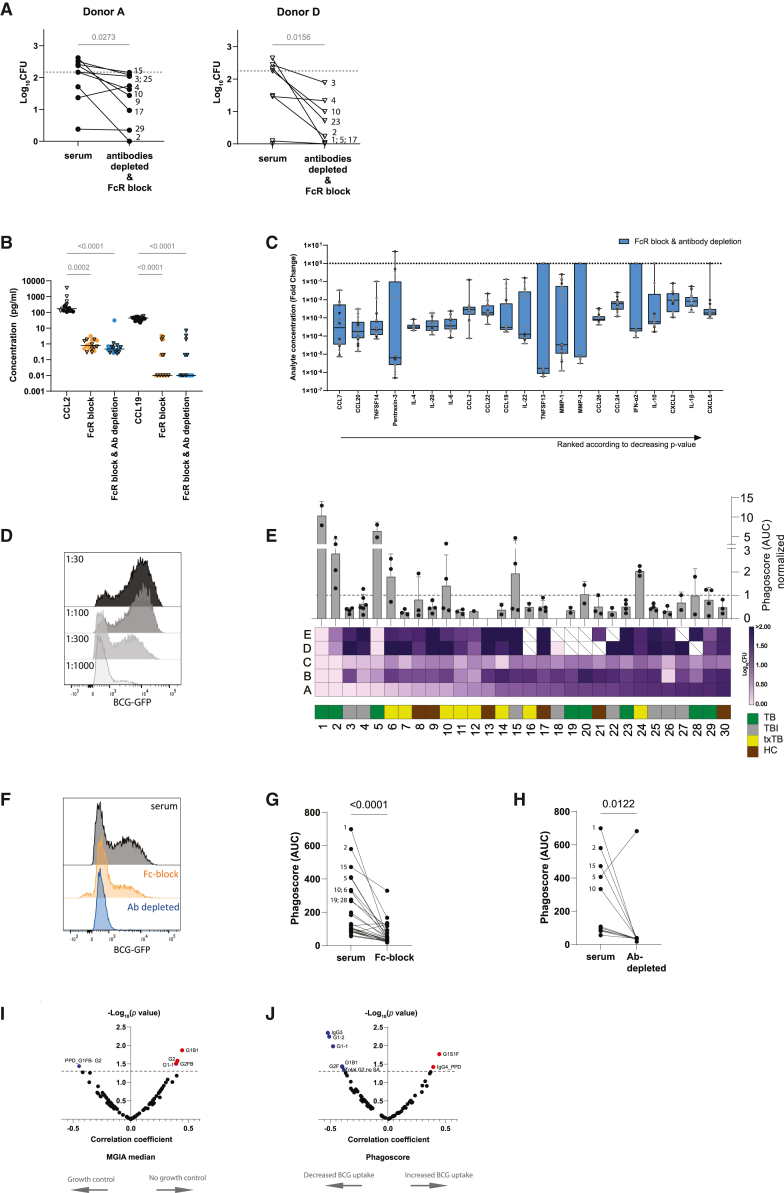
Phagocytosis capacity as mediator of mycobacterial growth control (A) MGIA was performed with FcR-block in combination with antibody depleted sera for sera of 5 TB disease (1, 2, 5, 23, and 29), 4 TB infected (3, 4, 15, and 25), 1 TB treated (10) and 2 healthy individuals (9 and 17) and results are shown as before after plots. Sera are numbered and the gray dotted line represents the inoculum. Statistical significance was tested by Wilcoxon paired analysis. (B) Soluble analytes were measured in supernatants of MGIA cocultures with and without Fc-block and antibody depletion. Concentrations for two example analytes, CCL2 and CCL19, are shown in a scatterplot with lines representing median. Dots represent results for PBMC donor A and open triangles PBMC D. For all analytes the difference between serum condition and the condition with FcR-block (orange) or with additional antibody depletion (blue) was tested by a Friedman test with Dunn’s multiple test correction. (C) Analytes for the condition of FcR-block and antibody depletion that reached significance were ranked on their *p* value and the fold change of the analyte concentration was plotted as boxes with median and whiskers at the 5–95 percentile. Black dots represent results for PBMC A and the open gray triangles PBMC D. (D) Phagocytosis of serum opsonized BCG-GFP by THP-1 cells at 1 h was assessed and serial dilutions of serum 1 are displayed. On the x axis the BCG-GFP signal is shown and dilutions 1:30, 100, 300, and 1000 are overlayed in gray shadings. (E) AUC of phagoscores (% BCG-GFP^+^ cells ∗ MFI of BCG-GFP^+^ cells)/MFI of THP-1 only control) were calculated for all sera (*n* = 30) and experiments, normalized against a reference serum and plotted as bar graphs (mean with SD) with each black dot representing a measurement. The bar graph was aligned with the intensity map (Figure 2B) to directly show the association between phagocytosis capacity and mycobacterial growth control. (F) Overlay histogram representing phagocytosis of serum opsonized BCG-GFP by THP-1 cells in the presence of FcR block (orange) and with antibody depleted serum (blue). (G) Phagoscores are plotted for sera without and with FcR block (*n* = 29) and (H) for sera vs. the antibody depleted sera (*n* = 12). Serum numbers are indicated for sera with the highest phagoscores. All tested conditions were analyzed by Wilcoxon test and *p* values are shown (I) Spearman’s rank correlation between MGIA result and available systems serology data (antibody concentrations and glycosylation) was assessed on the MGIA median result and the correlation coefficient r is plotted against the -log_10_(*p* value). Gray dotted line represents significance threshold (*p* value = 0.05). Red dots show positive correlation and blue dots negative correlation with MGIA results. (J) Correlation between phagoscore and results from systems serology were analyzed and plotted as in (I).

To further establish the importance of antibodies and FcRs, mycobacterial growth control was assessed comparing PBMC, with or without pre-incubation with FcR blocking reagent and compared to antibody depleted serum (with depletion efficiency visualized in Figure S2) for the 12 selected sera also shown in Figure 2. In both PBMCs A and D, an unexpected but significant CFU reduction was detected for conditions with FcR block and depleted antibodies (Figure 4A), which might reflect antibody-mediated Fc-receptor interactions as factor in mycobacterial growth control. This effect was not observed with FcR blockade only (Figure S3A). Either this blockade could have been incomplete or mycobacterial internalization was facilitated by ligation to mannose-, scavenger-, NOD- or Toll-like receptors. An alternative interpretation of these data could be that antibodies are detrimental to mycobacterial growth control, independent of FcR interactions. However, we consider this highly unlikely given the strong association with phagocytosis described in the following section and the necessity of monocytes to become infected with mycobacteria.

Supernatants of these cocultures had decreased concentrations for 58 out of 70 soluble analytes upon FcR blockade and seven more upon additional antibody depletion (65 out of 70). Examples for CCL2 and CCL19 are shown in Figure 4B. While a direct correlation between increasing levels of cytokines and chemokines with increasing mycobacterial growth control was shown in Figures 2C and 2D, here the observed CFU reduction coincided with significant decreased concentrations of cytokines/chemokines for both conditions tested. FcR block only reduced concentrations of 17 analytes significantly as shown by the fold change compared to the serum condition (Figure S3B and supplementary datafile). When besides FcR blocking also antibodies were depleted, 21 analytes showed significant decrease in concentrations (Figure 4C; supplementary datafile) with median fold changes over 10^5^. Thus CFU reduction with antibody depleted sera without an accompanying increase in cytokine and chemokine concentrations does not directly implicate control of mycobacterial growth but could also point to lack of immune activation through Fc-mediated interactions. As monocytes showed the most heterogeneous expression of FcRs and growth restriction indicated antibody-mediated effects, the monocytic cell line THP-1 was used to investigate FcR-mediated interactions. Phagocytosis of BCG-GFP by THP-1 cells was measured in serial serum dilutions by flow cytometry (Figures 4D, S4A, and S4B). While it is difficult to differentiate internalization from binding with this method, data depicted in Figure S4C shows for three sera of individuals with TB no CFU reduction upon gentamicin treatment, indicating the BCG-GFP signal measured is not the result of mycobacteria at the cell surface of the phagocytic cells. Subsequently, phagocytosis was expressed as normalized phagoscore next to the results of BCG growth control (Figure 4E). The three sera showing the best growth control, #1, 2, and 5, also demonstrated the highest capacity to enhance phagocytosis of BCG. All other sera were close or equal to normal reference serum. FcR blocking of THP-1 cells before addition of serum opsonized mycobacteria, partially blocked BCG phagocytosis both in percentage of BCG-GFP^+^ cells (49%–30%) and in decreased mean fluorescent intensity (2499–1493), resulting in lower integrated phagoscores (Figures 4F and 4G). A greater reduction of BCG phagocytosis was observed for sera depleted of antibodies, with only background levels of uptake remaining (Figures 4F and H). Antibody concentrations and isotype distributions for IgG, IgA, or IgM were similar for all sera (Figure S5A). Even the three sera with the best MGIA control and phagocytic capacity did not deviate from the group means, indicating that qualitative differences of the antibodies may contribute to the observed functional differences. In addition, glycosylation analysis was performed on Fc-IgG subclass level for the limited number of enriched antibodies samples derived from antibody depletions. Analysis of fucosylated structures revealed similar glycosylation profiles for the IgG subclasses within sera and galactosylation differences between sera (Figure S5B). Finally, correlation analysis between functional measures MGIA and phagocytic capacity to the known (antigen-specific) antibody concentrations and glycosylation features for the 30 sera tested was performed. A significant correlation was observed for MGIA with galactosylation structures G1B1, G1-1, G2, and G2FB on one side and PPD-specific G1FB-G2 on the other side, as displayed with correlation coefficient and log_10_(*p* value) for the growth control median value as a consolidated value among donors (Figure 4I). However, heterogeneity was observed with a variety of significant glycosylation features across donor PBMCs pointing to donor related capacity to control BCG growth possibly involving their FcRs (Figure S5C). Correlation to normalized phagoscore identified negatively correlating total IgG-Fc with galactosylated structures besides total IgG3 antibodies and positive associations with total IgG-Fc G1S1F structure and PPD-specific IgG4 (Figure 4J).

## Discussion

Sera from individuals with TB disease, TB infection or after completion of TB treatment were analyzed together with sera from HCs in an adapted *in vitro* MGIA. We demonstrated that sera could contribute to BCG growth control and this effect was reduced or abrogated when FcR on PBMCs were blocked and serum antibodies depleted, concomitantly resulting in lack of immune cell activation as detected by reduced chemokine and cytokine secretion. The magnitude of the observed growth control by serum varied from a strong contribution to little or no impact. All sera were tested in the same experiment on the same PBMC samples to rule out donor and inter-assay variability as much as possible. Sera capable of controlling BCG growth displayed enhanced phagocytic capacity compared with sera without growth control, pointing to an association of antibodies with FcR-mediated phagocytosis.

Currently, more insight into the role of the innate immune components, B cells and antibodies against infection with TB is accumulating. As previously shown in samples of the same cohort, B cells of active TB patients were dysfunctional in proliferative capacity and cytokine production, which were both restored in treated TB individuals.^10^ Likely, also other B cell related functions as production of antibodies could be impacted. Systems serology uncovered associations between different stages of TB disease and antigen-specific antibodies and/or antibody glycosylation features.^14^ To be able to correlate these findings with functional data on mycobacterial growth control we have adapted the mycobacterial growth assay to explore the capacity of sera to enhance growth control. Sera were diverse in their contribution to mycobacterial growth control *in vitro*, which was further augmented by differences in FcR expression profiles of the PBMC donors. The best-controlling sera were able to significantly decrease mycobacterial growth by more than 1 log in PBMCs from several donors. Combined antibody depletion and FcR block seemingly enhanced mycobacterial growth inhibition capacity, with argues against a beneficial contribution of antibodies in growth inhibition. However, a first prerequisite for killing of intracellular mycobacteria is the actual infection of the monocytes, which is abrogated upon antibody depletion as reflected by the phagocytosis data. Thus, we believe that the initially contradiction MGIA data of antibody depleted sera are the result of absent infection, rather than growth promoting antibody effects. Capacity to reduce CFU did not correlate to PPD or single antigen-specific antibody titers, possibly due to the high number of proteins expressed by *M.tuberculosis* that can potentially induce antibody responses for which PPD only represents roughly 5% of the total *Mtb* proteome. Besides PPD, only seven single *Mtb* antigens were assessed in the systems serology approach implicating that possible relevant antigen-specific antibodies were not detected thereby underpowering the correlation analysis.^28^^,^^29^^,^^30^^,^^31^

Many correlations between antigen-specific antibodies and TB disease groups have been described over the last decade^11^^,^^14^^,^^23^^,^^24^^,^^32^^,^^33^ but to our knowledge most analyses were based on comparing antigen or PPD-specific antibody titers, between disease groups or cohorts and few where related to the functional potential of these antibodies.^11^^,^^14^ Dijkman et al. demonstrated in non-human primates mucosal IgA to be a biomarker for mucosal delivered BCG induced protection to *Mtb*, but its function remains to be resolved.^34^ Comparing pre- and post-BCG vaccination samples, Chen et al. showed correlations of AM-specific IgG antibody titers post vaccination with increased phagocytic capacity and mycobacterial growth control at 4 weeks post-vaccination. Unfortunately, there was no direct evaluation of the AM IgG antibody function, as the MGIA was performed with PBMC cocultured with 10% normal pooled human serum and not the matched post-vaccination serum or plasma.^26^^,^^35^ A BCG vaccination study in non-human primates showed significant control of mycobacterial growth when PBMC and autologous sera were matched for time points.^36^ However, the effect on growth control was lost when sera from the post-vaccination time point (day 84) were exchanged with the pre-vaccination PBMC samples and vice versa, indicating that the serum in this study could not mediate control of mycobacterial growth by itself, even though the opsonization capacity was increased in post-vaccination samples.^36^ This could indicate a role for cellular interplay and more specifically the FcR repertoire on monocytes as the MGIA was performed in an autologous setting. This interdependence was excluded in the present study by use of common PBMCs, assessing sera as the only variable.

Cytokine and chemokine levels in culture supernatants indicated an activated state of immune cells in the MGIA. CCL2 for example, is known to regulate cellular processes and exhibits chemotactic activity for monocytes whereas CCL19 and CCL21 are both ligands for CCR7 and increased levels are measured during inflammation.^37^ Hypothetically, these chemokines may contribute to TB control by attracting monocytes to the site of infection via CCL2 and antigen-engaged B cells and central memory T cells via CCL19 and CCL21, respectively. When FcRs were blocked, analyte levels decreased drastically, sometimes even to undetectable levels in all sera, suggesting mycobacterial uptake to be essential for immune cell activation. When on top of the FcR block sera were depleted for antibodies, the cytokine and chemokine levels decreased even further as well as mycobacterial growth, measured by CFU, indicating that antibodies in the *in vitro* functional assay contribute, possibly via triggering of FcR.

Diversity in FcR expression levels and patterns was observed, primarily on monocytes. Altered FcR expression and function is seen in patients with autoimmune diseases like Systemic lupus erythematosus and Hashimoto’s thyroiditis and in infectious diseases like HIV^38^ and Hepatitis B virus, the latter e.g., revealing decreased expression of FcyRIIb in liver tissue samples from chronic hepatitis B patients, that correlate to disease severity.^39^ A study by Sutherland et al. indicated FcγRI as an RNA transcription biomarker that could discriminate active TB cases from TB-infected individuals^40^ and elevated levels of FcyRIIIa were seen in TB-infected individuals compared to uninfected controls, indicating that antibodies may act differently in these individuals.^41^ These studies correlate FcR expression patterns to disease outcome, which can also contribute to the efficacy of the immune response towards BCG or *Mtb*. The heterogeneous and diverse FcR surface expression seen here, between PBMCs and cell subsets, may contribute to variations in antibody binding between donors and cell types and subsequent growth control. However, the importance of the individual FcRs in relation to growth control should be studied in more detail and should, for example, include specific FcR blockage.

Opsonophagocytosis is one of the main functions of antibodies and it has been shown that this Fc-mediated effector function is increased in animal and human studies on (antigen-specific) antibodies of TB patients, TBI and BCG vaccinees.^11^^,^^23^^,^^24^ In our study, sera with the highest capacity to control mycobacterial growth also induced the highest phagocytic capacity of BCG, but this was not related to higher levels of IgG, IgA, or IgM. Studies on Fc-modifications have shown that there is a direct Fc-mediated effect on immune cell activation by glycosylation variation.^11^^,^^15^^,^^17^ Recently, Pongracz et al. studied the total blood N-glycome in a meta-analysis to unravel disease associating glycan signatures with respect to the complexity of the glycomic dimension and showed that in several cancer types, metabolic, infectious and autoimmune diseases the glycan abundance reveals diverse but sometimes overlapping signatures under different pathological conditions.^42^ However, a common inflammatory response underlies alterations of glycosylation forms.^15^^,^^42^ Interestingly, in our study, most of the glycosylation features that correlate to “no growth control” are total IgG-Fc structures that are not fucosylated, thereby likely having a strong interaction with FcRs and potentially competing for binding with antigen-specific antibodies.^43^^,^^44^ These data emphasize the importance of continuing these explorations and looking beyond antibody titers and characterize antibody specificity, glycosylation and other post-translational modifications from a functional perspective.

In this study we have described the functional contribution of TB immune sera, covering the whole breadth of antibody classes and spectrum of TB, to control mycobacterial growth in an adapted MGIA. Sera were compared within single PBMC donors, thereby studying serum components as the only variable of BCG growth control. We observed qualitative differences between sera tested on the same donor PBMCs and have shown that sera modulate growth control and affect the phagocytic capacity mediated by antibody-FcR interactions.

### Limitations of the study

This study was limited in terms of the availability of demanding serum quantities for Fc-blocking and antibody depletion experiments. Not all sera yielded PPD-specific glycosylation profiles and unfortunately, upon analysis this information was missing for several of our most interesting sera with relation to growth control and phagocytosis. The MGIA was performed with BCG and not with virulent *Mtb*. The contribution of *Mtb*-specific antibody interactions was therefore limited to antibodies cross reactive with BCG. While this is a limitation to the study, it was shown by Venkataraman et al. that in a whole blood MGIA results between BCG and *Mtb* significantly correlate.^45^ Furthermore, this study analyzed total Fc-block and no data are available for more detailed and specific FcR blocking reagents, which could also identify cellular interactions in relation to the FcR profiles and Fc-mediated effector functions. To test this in the functional MGIA it would require considerable amounts of sera and PBMCs, which were limited. Phagocytosis assays were only performed with the THP-1 monocytic cell line which has the limitation of very low expression of FcγRIII, such that these interactions, with the available antibody characteristics, were not studied; phagocytosis by monocytes would be a valuable addition in further research.

## Resource availability

### Lead contact

Requests for further information should be directed to and will be fulfilled by Krista E. van Meijgaarden (k.e.van_meijgaarden@lumc.nl).

### Materials availability

This study did not generate new unique reagents.

### Data and code availability

• All data generated or analyzed during this study are included in this published article. Raw data from figures have been deposited at Mendeley Data and are publicly available as of the date of publication. Accession numbers are listed in the key resources table.
• This paper does not report original code.
• Any additional information required to reanalyze the data reported in this paper is available from the lead contact upon request.

## Acknowledgments

The authors are grateful to all the patients, nurses, and physicians who helped to perform this study at the National Institute for Infectious Diseases, Rome, Italy, and Alessandra Aiello, from the Department of Epidemiology and Preclinical Research at the National Institute for Infectious Diseases, Rome, Italy, for critically reading the manuscript.

This work was supported by funding from the 10.13039/100010661European Commission (EC) Horizon2020, FP7 NEWTBVAC project contract no. LSHP-CT-2003-503367, TBVAC2020 (grant agreement 643381), the 10.13039/501100005039Leiden University Medical Center, and the National Institute for Infectious Diseases - IRCCS L.Spallanzani, Rome, Italy; Linea 4 - Ricera Corrente, Progetto 2 by the 10.13039/501100003196Italian Ministry of Health.

## Author contributions

K.E.v.M.: conceptualization, investigation, methodology, formal analysis, visualization, and writing-original draft; P.S.G.: writing – review and editing; W.W.: investigation; D.G. and F.P.: resources; M.W.: supervision; T.H.M.O.: supervision and funding acquisition; S.A.J.: conceptualization, visualization, writing – review and editing, and supervision.

## Declaration of interests

The authors declare that they have no competing interest.

## STAR★Methods

### Key resources table

**Table:** 

REAGENT or RESOURCE	SOURCE	IDENTIFIER
**Antibodies**		

CD3 - APC-Cy7 (clone SK7)	BD Biosciences	Cat# 557832; RRID:AB_396890
CD4-AlexaFluor700 (clone RPA-T4)	BD Biosciences	Cat# 557922; RRID:AB_396943
CD8-PE-Cy5 (clone RPA-T8)	BD Biosciences	Cat# 555368; RRID:AB_395771
CD19-BV605 (clone SJ25C1)	BD Biosciences	Cat# 562654; RRID:AB_2909453
CD56-PE-Cy7 (clone B159)	BD Biosciences	Cat# 557747; RRID:AB_396853
CD16-PE-CF594 (clone 3G8)	BD Biosciences	Cat# 562293; RRID:AB_11151916
CD32-PE (clone 3D3)	BD Biosciences	Cat# 552884; RRID:AB_394513
CD64-FITC (clone 10.1)	BD Biosciences	Cat# 555527; RRID:AB_395913
CD89-APC (clone A59)	Biolegend	Cat# 354106; RRID:AB_2565257
CD14-BV786 (clone M5E2)	BD Biosciences	Cat# 563698; RRID:AB_2744287

**Bacterial and virus strains**		

BCG-GFP	Leiden University Medical Center	

**Critical commercial assays**		

BACTEC MGIT tubes	Becton Dickinson	245122
Panta/Enrichment MGIT tubes	Becton Dickinson	245124
Bio-Plex Pro human Chemokine panel 40-plex	BioRad Laboratories	#171AK99MR2
Bio-Plex Pro Human Inflammation panel 37-plex	BioRad Laboratories	#171AL001M

**Deposited data**		

Data belonging to this manuscript	Mendeley Data: https://doi.org/10.17632/2kn7kdzmw5.1	https://doi.org/10.17632/2kn7kdzmw5.1

**Experimental models: Cell lines**		

THP-1 cell line	ATCC	TIB202

**Software and algorithms**		

GraphPadPrism	Dotmatics	v10.2.3
Flowjo	BD Biosciences	v10.8.0
BioPlex manager v6.2	BioRad Laboratories	v6.2
EpiCenter	Becton Dickinson	

**Other**		

Roswell Park Memorial Institute medium I - dutch modification	Gibco - life sciences	10575573
Iscove’s modified Dulbecco’s Medium	Gibco - life sciences	11514456
Glutamax	Gibco - life sciences	10860311
FcR blocking reagent	Innovex BioSciences, Gentaur	NB335
SpinTrap Protein G column	Cytiva	28903134
SpinTrap Protein A column	Cytiva	28903132
anti-human Fc IgA	Bethyl laboratories	Cat# A80-102A; RRID:AB_67044
anti-human Fc IgM	Bethyl laboratories	Cat# A80-100; RRID:AB_67078
anti-human Fc IgG	Bethyl laboratories	Cat# A80-104; RRID:AB_67060
anti-goat IgA - HRP	Bethyl laboratories	Cat# A80-102P; RRID:AB_67047
anti-goat IgM - HRP	Bethyl laboratories	Cat# A80-100P; RRID:AB_67082
anti-goat IgG - HRP	Bethyl laboratories	Cat# A80-104P; RRID:AB_67064
TMB chromogen solution	ThermoFisher	10647894
BACTEC MGIT960 system	Becton Dickinson	
LSRFortessa	Becton Dickinson	
Bio-plex 200 system	BioRad laboratories	
iMark ELISA reader	BioRad laboratories	

### Experimental model and study participant details

Sera ( n= 8) and plasma ( n= 24) were collected from 30 well-characterized individuals with active TB disease (TB) (n=8), latent infection (TBI) (n=8), treated TB (txTB) (n=8) and healthy controls (HC) (n=6) that were part of a larger cohort previously described^10^^,^^14^ and recruited at the National Institute of Infectious Diseases in Rome, Italy (demographic information in supplementary data file). Serum and plasma samples will be collectively referred to as sera. Sera were not collected and processed through a cold chain, ensuring all sample handling below 4°C, to preserve complement activity. Briefly, individuals with pulmonary TB disease were microbiologically diagnosed^46^ and received standard care treatment (isoniazid, rifampicin, ethambutol and pyrazinamide for 2 months, followed by isoniazid, rifampicin for 4 additional months). Blood and serum collection was performed within the first week of treatment. Individuals treated for TB disease had received the six month standard treatment and were culture negative at two and six months of therapy. Sera were collected a few months to several years after completion of treatment. Individuals with latent infection were exposed to patients with active TB disease, within six months before blood collection, and were positive for QuantiFERON TB Gold In Tube test (QFN) (Qiagen, Germany) and remained without TB disease symptoms for 8 to 10 years following the blood donation and plasma or serum collection. Healthy individuals were scored QFN negative and all individuals were HIV negative. All samples were pseudo-anonymized by laboratory codes and serum or plasma samples were collected for research purposes. Samples included in this study were selected based on availability. Heatmaps were generated in Morpheus software (software.broadinstitute.org).

#### Ethics statement

This study was approved by the Ethical Committee of the L. Spallanzani National Institute of Infectious Diseases (INMI), with approval numbers 02/2007 and 72/2015. Informed written consent was required to participate in the study and was obtained before collecting blood samples.

### Method details

#### Mycobacterial growth inhibition assay

PBMCs were isolated by Ficoll (Pharmacy LUMC, the Netherlands) density gradient separation of buffycoats from healthy blood bank donors (Sanquin, Bloodbank, the Netherlands) and were frozen in RPMI (Gibco Life Sciences, ThermoFisher Scientific Inc, Bleiswijk, The Netherlands) supplemented with 10% FBS (Corning Life Science, Amsterdam, the Netherlands) and 10% DMSO (Merck Life Science, Amsterdam, the Netherlands) and stored in liquid nitrogen until further use. These cryopreserved PBMCs were used as cellular source for the adapted Mycobacterial Growth Inhibition assay and do not in any way relate to the serum donors studied in this manuscript. PBMCs were thawed and rested for 2 hours in RPMI, supplemented with Glutamax (Gibco) and 10% FBS (Corning) (R10) in the presence of 25U/ml Benzonase at 3-5x10^6^ cells/ml (Merck). During this resting step live BCG (strain P3) from well controlled frozen stocks was thawed, diluted to approximately 100-300 CFU per 300 μl and incubated with 60 μl serum (10% of final volume) for at least 30 minutes prior to addition of the PBMCs. After resting, PBMCs were washed twice in RPMI, counted with a Casy cell counter (Roche, Woerden, The Netherlands) and 300 μl of 3.3x10^6^ PBMCs was added to each sample and cocultured for 96 hours on a rotator in a 37°C, 5% CO_2_ humidified incubator.^47^ All samples were run in duplicate. After 4 days, 60 μl supernatant was collected and stored for further analysis by Luminex multiplex assays. The remaining 600 μl was resuspended and transferred to PANTA/Enrichment supplemented MGIT tubes (Becton Dickinson, Erembodegem, Belgium) and placed in a BactecMGIT 960 system (BD) until time to positivity (TTP) was reached. Samples positive within the first 50 hours of cultures were considered contaminated and excluded from further analysis. Before removal from the BactecMGIT incubator all tubes were visually checked for mycobacterial growth. To calculate the mycobacterial growth inhibition a 10x serial dilution series standard curve of the BCG stock was generated in all assays for both the MGIT tubes and the corresponding TTP, as well as plated on Middlebrook 7H10 agar plates, supplemented with 10% OADC (BD) for CFU determination. CFU’s were converted to log_10_CFU and plotted against the TTP. Linear regression analysis was applied (Graphpad Prism software v9.3) and all samples were transposed and data are plotted as log_10_ CFU.

For FcR blocking experiments, PBMCs were rested in the presence of Benzonase for 2 hours, washed, counted and incubated at room temperature (RT) for an additional hour at 1x10^6^ cells in 500 μl FcR block (universal Fc Receptor Blocker, Innovex Biosciences, Gentaur, Eersel, the Netherlands), washed and added to the corresponding samples.

#### Antibody depletion

To study the effect of antibodies on mycobacterial growth and phagocytosis, sera (300 μl/column) were depleted from antibodies by rotating incubation, for at least 2 hours on SpinTrap Protein G columns (Cytiva, VWR international, the Netherlands). According to the manufacturer’s instructions depleted fractions were retrieved by spinning the columns for 1 minute at 100x g and flow through was transferred to the sequential Protein A column for further depletion of remaining antibodies. After the additional 2 hour incubation and spinning, the antibody depleted fractions were aliquoted and stored at -20°C. The immunoglobulins that remained on the protein G and A columns were eluted in 400 μl 20 mM glycine buffer pH3 and neutralized by 40 μl 1M Tris pH9.7 before glycosylation analysis was performed.

#### Immunoglobulin quantification ELISA

Serum depleted fractions were checked for IgA, IgM and IgG concentrations by ELISA (Figure S2). Maxisorp plates (NUNC) were coated with 100 μl/well capture antibody anti-human Fc IgA, IgM or IgG, (10 μg/ml, Bethyl Laboratories, SanbioBV, Uden, the Netherlands) in sodium carbonate coating buffer pH9.6 for 1 hour at RT, washed and blocked with 200 μl PBS/ 1% BSA/50mM tris pH8.0 (PBT) for 30 minutes, RT. Samples and standards were diluted in 10x and 2x serial dilutions respectively in PBT/0.05%Tween20 (PBTT), 100 μl was added to the coated wells and incubated for 1 hour at RT. Plates were washed three times and incubated for 1 hour with 100μl HRP conjugated anti-goat IgA, IgM or IgG- 50 ng/ml (Bethyl Laboratories), washed again and developed with TMB chromogen solution (ThermoFisher). Colour reaction was stopped with 1 M H_2_SO_4_ and OD_450nm was measured on a BioRad iMark reader.

#### Luminex multiplex assays

Multiplex bead assays were performed according to manufacturer’s instructions. Supernatants collected from the 96 hr BCG infection cocultures were measured with the Bio-Plex Pro human Chemokine panel (40-plex, #171AK99MR2), and the Bio-Plex Pro Human Inflammation panel (37-plex, #171AL001M) (Bio-Rad Laboratories, Veenendaal, the Netherlands). Samples were diluted 1:2 in sample Diluent HB and run as single measurement with the streptavidin PE (1:200, Becton Dickinson) detection label. Samples were acquired on a Bio-Plex 200 system and analysed with Bio-Plex manager software v6.2. In total 73 unique soluble analytes were measured per sample.

#### Flow cytometry

FcR expression was determined by surface staining and flow cytometry. PBMCs were washed in PBS and stained with the vivid live dead stain (Invitrogen, ThermoFisher), 5 μl 1:40 per 1x10^6^ cells in the dark for 10 minutes. The following surface markers were added in a total volume of 50 μl PBS/0.1%BSA per sample including Brilliant Violet Stain buffer (Becton Dickinson). CD3-APC-Cy7 (clone SK7), CD4-AlexaFluor700 (clone RPA-T4), CD8-PE-Cy5 (clone RPA-T8), CD19-BV605 (clone SJ25C1), CD56-PE-Cy7 (clone B159), CD16-PE-CF594 (clone 3G8), CD32-PE (clone 3D3), CD64-FITC (clone 10.1) and CD14-BV786 (clone M5E2) (all Becton Dickinson Biosciences), CD89-APC (clone A59) (Biolegend, UK). Samples were acquired on a LSRFortessa (BD) and analyzed with Flowjo software v9.7 (Treestar Inc). Gating strategy is provided in the supplementary information (Figure S1).

#### Phagocytosis assay

THP-1 cells were cultured in Iscove’s modified Dulbecco’s Medium supplemented with glutamax (GIBCO, life sciences) and 10% FBS (Corning). Phagocytosis of BCG-GFP (generated at our laboratory, strain P3) was measured by flowcytometry. BCG-GFP (5x10^5^ bacteria/sample) were incubated in a 96well round bottom plate with 50 μl sera in the following dilutions 1:15, 1:50, 1:150 and 1:500 for 2 hours before 5x10^4^ THP-1 cells/sample were added in 50 μl and cocultured for 1 hour at 37°C in a 5% CO_2_ humidified incubator. Following incubation, samples were washed with PBS/0.1% BSA, fixed with 1% paraformaldehyde (LUMC pharmacy, the Netherlands) and 10^4^ THP-1 events were acquired on a FACSLyric (BD). Gating strategy is provided in the Figure S4). Phagocytosis in the presence of FcR blocking was performed by pre-incubation of 1x10^6^ THP-1 cells with 500 μl of the universal FcR blocking reagent (Innovex) for 1 hour before addition of opsonized BCG-GFP. For each sample and dilution the MFI of the phagocytosed BCG-GFP was determined in addition to the % of BCG-GFP positive cells and an integrated phagoscore was calculated ((% BCG-GFP^+^ cells ∗ MFI BCG-GFP^+^ cells)/MFI of THP-1 control cells). Furthermore for all sera the AUC of the phagoscore was determined using Graphpad Prism software by plotting the phagoscore (Y-axis) and the dilution (X-axis) for comparative analysis of sera and conditions. All phagocytosis experiments were performed in 3 to 5 independent experiments based on the availability of the sera and a reference serum was used to normalize data. A phagocytosis control experiment to differentiate internalization and surface binding of BCG-GFP was performed for three sera from individuals with a history of TB. Upon THP-1 incubation of opsonized BCG-GFP, samples were split in two and half were treated with gentamycin (Gibco Life Sciences, ThermoFisher Scientific Inc, Bleiswijk, The Netherlands) (30 μg/ml) for 15 minutes to kill all extracellular mycobacteria. Samples were washed twice and lysed before transferring to supplemented MGIT indicator tubes until time to positivity was reached.

#### Glycosylation analysis

After antibody depletion, total IgGs from both flowthroughs were enriched using protein G Sepharose Fast Flow 4 beads (GE Healthcare) as described,^48^ or using protein G coated Maxisorp NUNCImmuno plate (both Thermo Fisher Scientific) following by the GlYcoLISA method,^49^ whereas the specific IgG was affinity-captured using purified protein derivative (PPD; AJVaccines, Copenhagen, Denmark) coated Maxisorp plate. The eluted antibodies were subjected to tryptic digestion to obtain IgG glycopeptides, as described.^48^ IgG Fc glycopeptides were detected with an Impact quadrupole time-of-flight mass spectrometer (Bruker Daltonics) following separation using an Ultimate 3000 highperformance liquid chromatography (HPLC) system (Thermo Fisher Scientific) equipped with a nanoBooster. Ionization was enhanced by applying acetonitrile-doped nebulizing nitrogen gas at 0.2 bar. Profile spectra were recorded in an m/z range from 550 to 1800 with a frequency of 1 Hz. Based on accurate mass and retention time, IgG Fc glycopeptides were assigned. The relative quantification of IgG glycopeptide signals was performed using LaCyTools. A normal plasma frozen pool from a minimum of 20 healthy controls (VisuConF, Affinity Biologicals, Hamilton, Canada) was used as a positive quality control for the assay.

### Quantification and statistical analysis

For statistical analysis Graphpad Prism software v10.2 was used. Group comparison were tested by Kruskal-Wallis with Dunn’s multiple test correction**;** Friedman test with Dunn’s multiple test correction was used for analysis of the soluble analytes and the different conditions. Paired samples were assessed by Wilcoxon tests. Correlations were tested for significance by Spearman’s rank testing. All statistical details of the experiments can be found in the figure legends including the statistical tests used, number of samples included and definitions of center and dispersion and precision measures. A significance level of p < 0.05 was set. Heatmaps were generated using hierarchical clustering on rows and columns with Euclidean distance and average linkage method with resulting dendrograms using Morpheus software (Broad Institute) with relative colour scaling within each row. Flow cytometry analysis was performed using Flowjo v9.7 (Treestar inc.).
