# Supplementary material for: Sera contributing to mycobacterial growth restriction in vitro display enhanced Fc-mediated phagocytosis
Source: iScience. 2025 Apr 23;28(5):112504. doi: 10.1016/j.isci.2025.112504 (PMC12127566; doi:10.1016/j.isci.2025.112504)
Supplement: Document S1. Figures S1–S5 [file mmc1.pdf]

iScience, Volume *28*

Supplemental information

Sera contributing to mycobacterial growth restriction *in vitro* display enhanced Fc-mediated phagocytosis

Krista E. van Meijgaarden, Patricia S. Grace, Wenjun Wang, Delia Goletti, Fabrizio Palmieri, Manfred Wuhrer, Tom H.M. Ottenhoff, and Simone A. Joosten

Supplementary Figure 1

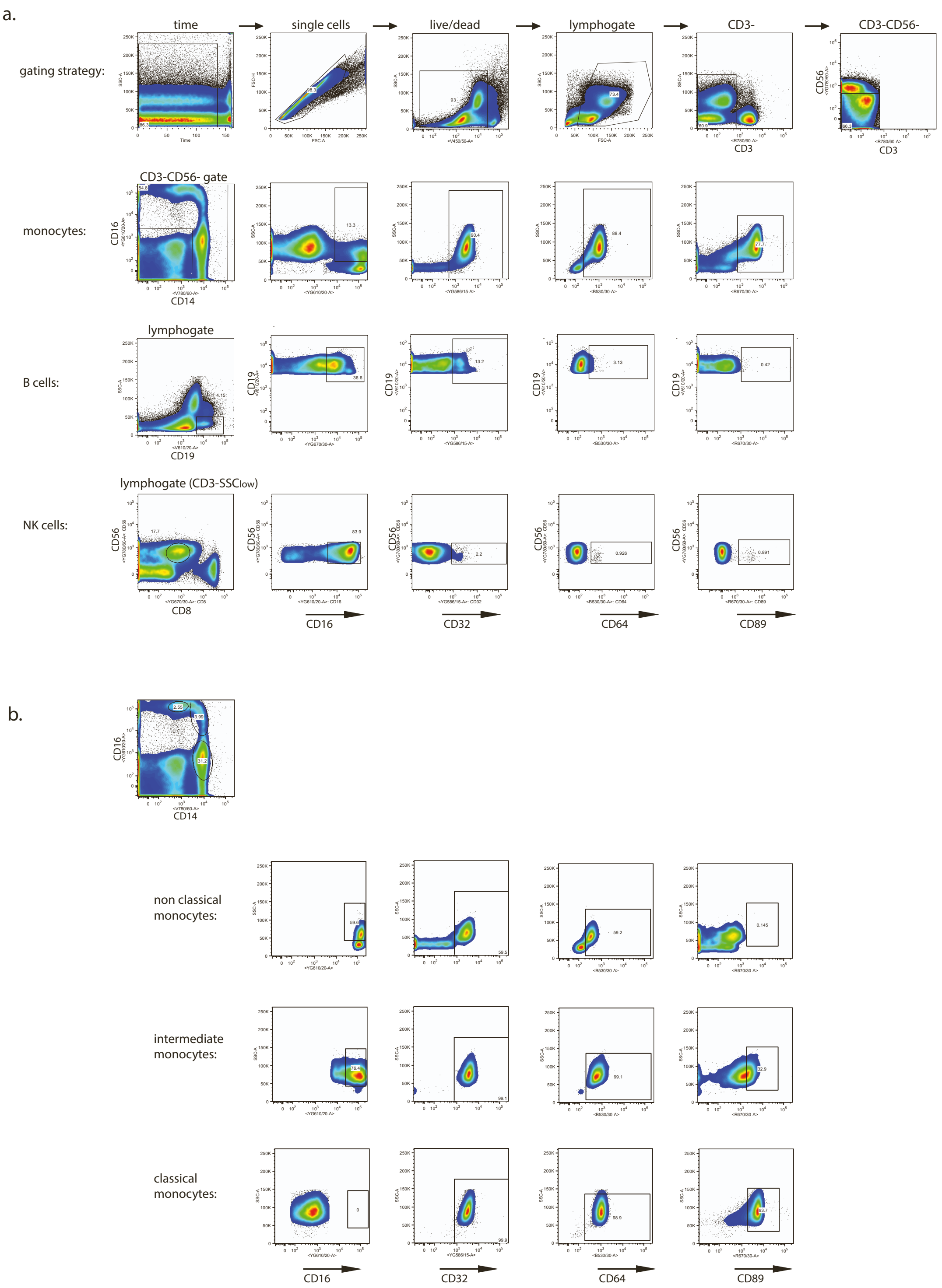

Supplementary Figure 1: Gating strategies of flow cytometry analysis of FcRs and phagocytosis assays. Supplementary Figure 1a shows the gating strategy of the Fc-receptor analysis on monocytes, NK cells and B cells; the monocytes subset analysis in supplementary Figure 1b.

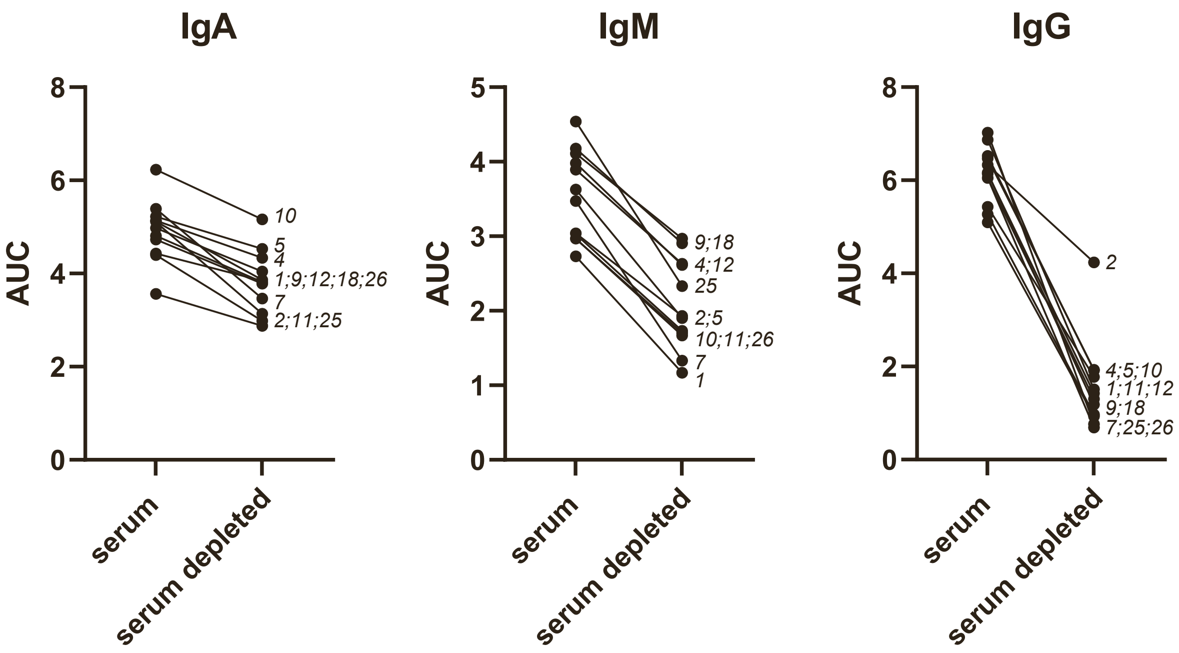

Supplementary Figure 2: Sera depleted for antibodies. Before-after charts for sera depleted for IgA, IgM and IgG by protein A and G spin columns and as measured by ELISA, showing the reduction of the antibody per sample. Sera were on average depleted of 22% of IgA, 42% IgM and 75% of their IgG.

Supplementary Figure 3

a.

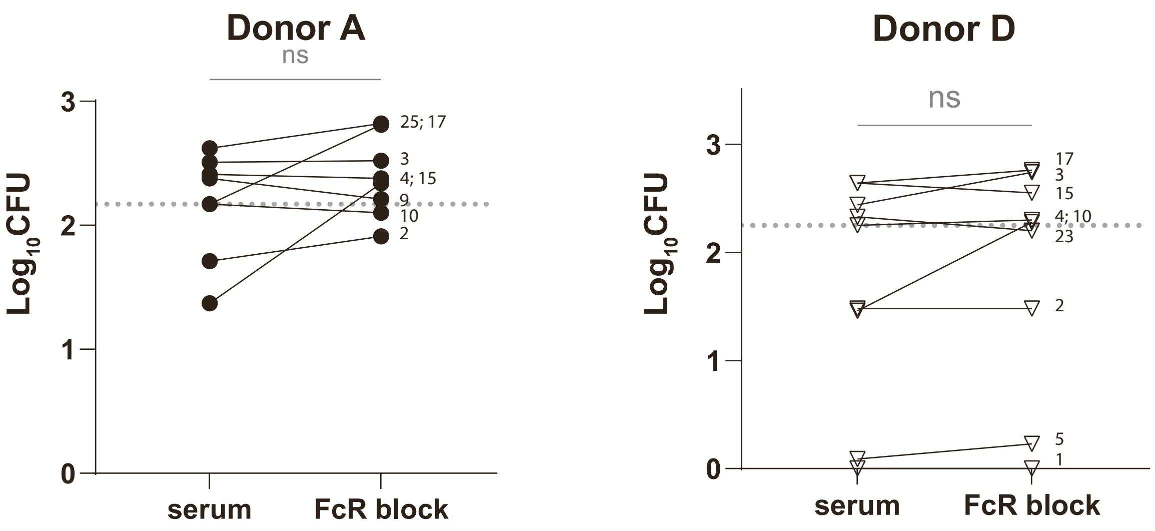

b.

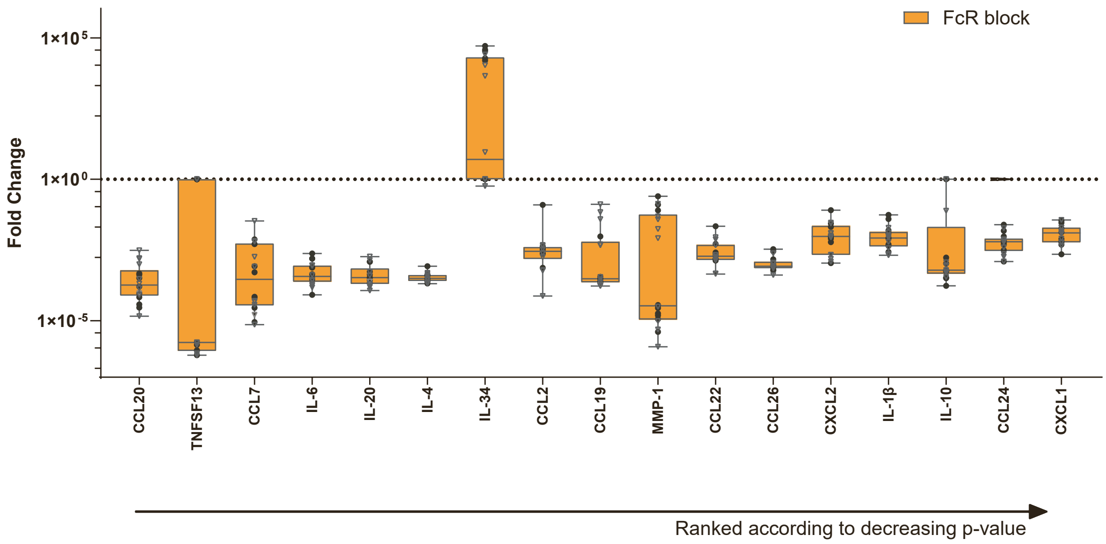

Supplementary Figure 3: FcR blocking in mycobacterial growth inhibition assay. (a) MGIA was performed with FcR-block for sera of 5 TB disease (1; 2; 5; 23; 29), 4 TB infected (3; 4; 15; 25), 1 TB treated (10) and 2 healthy individuals (9; 17) and results are shown as before after plots. Sera are numbered and the grey dotted line represents the inoculum. Statistical significance was tested by Wilcoxon paired analysis. **(b)** Analytes for the condition of FcR-block and antibody depletion that reached significance were ranked on their p-value and the fold change of the analyte concentration was plotted as boxes with median and whiskers at the 5-95 percentile. Black dots represent results for PBMC A and the open grey triangles PBMC D.

Supplementary Figure 4

a.

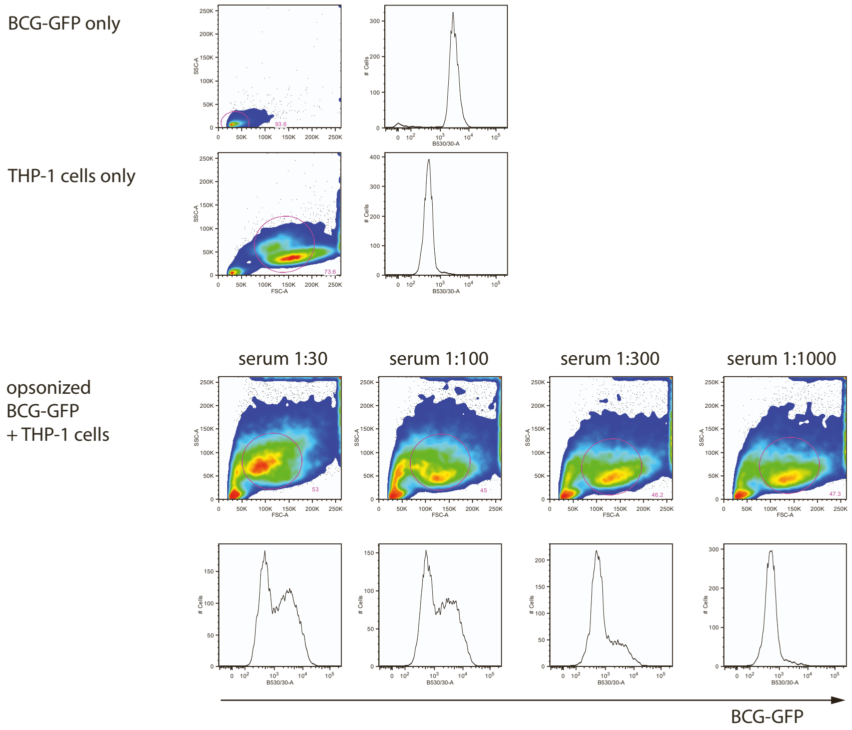

b.

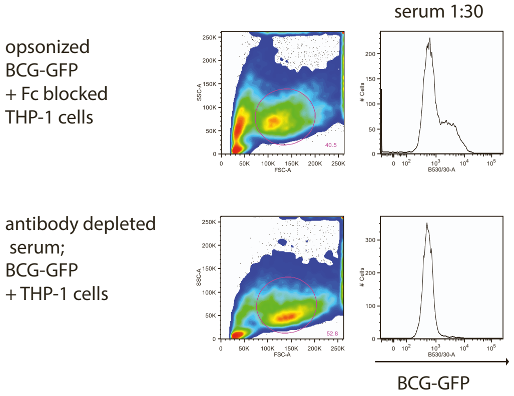

c.

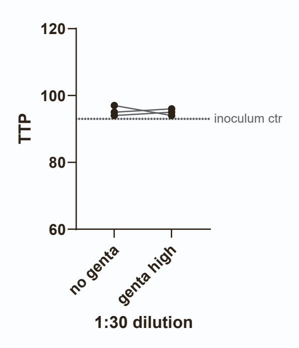

Supplementary Figure 4: Flow cytometry phagocytosis assays. (a,b) gating strategy for flow cytometric analysis of phagocytic capacity by THP-1 cells. (c) Control experiment to differentiate internalization and surface binding of BCG-GFP. Phagocytosis was performed as described for three sera from individuals with a history of TB. Upon THP-1 incubation of opsonized BCG-GFP, samples were treated with gentamicin, washed, lysed and transferred to MGIT indicator tubes. The least diluted sample with the highest concentration of antibodies was tested and shown in the before-after plot with the gentamicin conditions on the x-axis. Time to Positivity was determined and plotted on the y-axis. Dotted line represents the inoculum control.

Supplementary Figure 5

a.

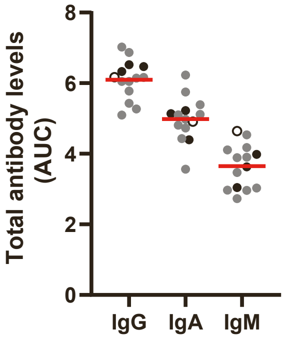

b.

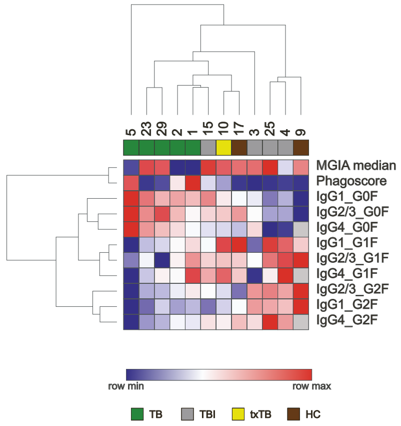

c.

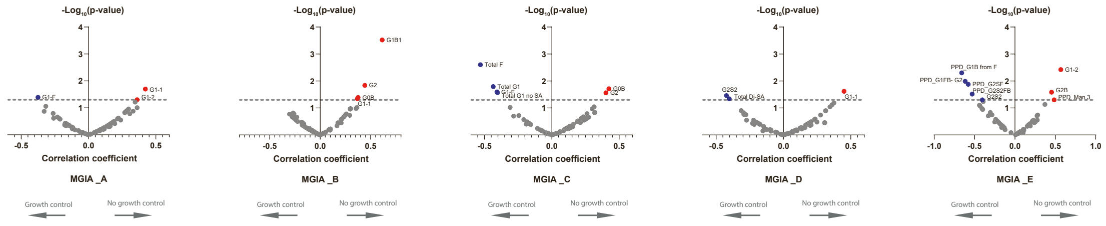

Supplementary Figure 5 : Total antibody levels. **(a)** Antibody levels as measured by ELISA for IgG, IgA and IgM, in black the 3 sera with highest phagoscore, open circle is reference serum. **(b)** Overview of functional data and IgG subclass fucosylated galactosylation profiles as analysed for the subset of samples included in the Fc-blocking and antibody depletion experiments (n=12). Heatmap was generated using hierarchical clustering on rows and columns with Euclidean distance and average linkage method with resulting dendrograms using Morpheus software (Broad Institute). Red to blue colour-scaling is relative within each row and represents the row minimum to maximum values of each feature indicated. Columns represent the individual sera. **(c)** Spearman's rank correlation between MGIA result and available systems serology data (antibody concentrations and glycosylation) was assessed per PBMC A to E and the correlation coefficient r is plotted against the -log$_{10}$(P-value). Grey dotted line represents significance threshold (P-value = 0.05). Red dots show positive correlation and blue dots negative correlation with MGIA results.
